# Analysis of Flavonoids and the Flavonoid Structural Genes in Brown Fiber of Upland Cotton

**DOI:** 10.1371/journal.pone.0058820

**Published:** 2013-03-19

**Authors:** Hongjie Feng, Xinhui Tian, Yongchang Liu, Yanjun Li, Xinyu Zhang, Brian Joseph Jones, Yuqiang Sun, Jie Sun

**Affiliations:** 1 The Key Laboratory of Oasis Eco-agriculture, Agriculture College of Shihezi University, Shihezi, China; 2 Faculty of Agriculture and Environment, University of Sydney, Sydney, Australia; 3 College of Life and Environmental Science, Hangzhou Normal University, Hangzhou, China; New Mexico State University, United States of America

## Abstract

**Backgroud:**

As a result of changing consumer preferences, cotton (*Gossypium Hirsutum* L.) from varieties with naturally colored fibers is becoming increasingly sought after in the textile industry. The molecular mechanisms leading to colored fiber development are still largely unknown, although it is expected that the color is derived from flavanoids.

**Experimental Design:**

Firstly, four key genes of the flavonoid biosynthetic pathway in cotton (*GhC4H, GhCHS, GhF3′H*, and *GhF3′5′H*) were cloned and studied their expression profiles during the development of brown- and white cotton fibers by QRT-PCR. And then, the concentrations of four components of the flavonoid biosynthetic pathway, naringenin, quercetin, kaempferol and myricetin in brown- and white fibers were analyzed at different developmental stages by HPLC.

**Result:**

The predicted proteins of the four flavonoid structural genes corresponding to these genes exhibit strong sequence similarity to their counterparts in various plant species. Transcript levels for all four genes were considerably higher in developing brown fibers than in white fibers from a near isogenic line (NIL). The contents of four flavonoids (naringenin, quercetin, kaempferol and myricetin) were significantly higher in brown than in white fibers and corresponding to the biosynthetic gene expression levels.

**Conclusions:**

Flavonoid structural gene expression and flavonoid metabolism are important in the development of pigmentation in brown cotton fibers.

## Introduction

Naturally colored cotton has innate color in the fiber. With a growing demand amongst consumers for environmentally benign products, naturally colored cotton, which can be used with little or no processing and dying steps, is becoming increasingly attractive to textile industries [1]–[3]. Unfortunately, however, only brown and green naturally colored varieties are currently available, restricting the development of naturally colored cotton textile market. It may be possible to increase the diversity of available naturally colored fibers using conventional breeding, however, this approach is limited by a lack of understanding of the process of cotton fiber color formation. In order to progress the integration of new natural colors into cotton, it is imperative that guidance is provided to molecular breeding programs. The fundamental requirement for this is an exploration and understanding of the molecular basis of pigment synthesis and deposition in cotton fibers.

Several genetic loci potentially involved in the process have been identified. A major loci with an incomplete dominant inheritance pattern for cotton fiber color formation has been identified [4]–[5]. It has also been reported that two or more loci are important for the inheritance of cotton fiber color [6]. Zhan et al. [5] showed that a pair of incomplete dominant loci controlled fiber color formation, and that there are several ‘minor loci’ that play additional roles. Shi et al. [7] concluded that green fiber was dominant to brown fiber, while brown fiber was dominant over white, and that there are genetic interactions between lint and fuzz color genes. Pure white fiber can be obtained by separating and extracting pigments from the colored cotton fiber, indicating that the pigment is independent and does not co-exist with the cellulose in the colored cotton fibers [8]–[9]. Extraction, washing and color reaction experiments indicated that pigments in brown fibers belong to the flavonoid group [10]. Whereas these studies have given us important clues, the precise molecular basis of pigment synthesis and deposition in cotton is still unknown and further studies are required before there is sufficient understanding to begin the process of introgressing novel color modifications into elite cotton varieties.

**Figure 1:**
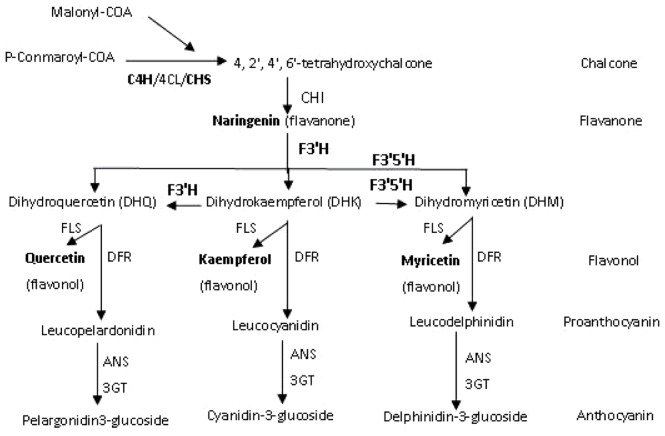
Schematic representation of the flavonoid biosynthetic pathway modified from [24]–[26]. C4H: Cinnamate-4-hydroxylase; 4CL: 4-Coumarate acyl-COA ligase; CHS: chalcone synthase; CHI: chalcone isomerase; F3′H: flavonoid-3′-hydroxylase; F3′5′H: flavonoid-3′5′-hydroxylase; FLS: flavonol synthase; DFR: dihydroflavonol reductase; ANS: Anthocyanidin synthase; 3GT: 3-O-glucosyltransferase.

In plants, three major classes of flavonoids (anthocyanins, proanthocyanidins and flavonols) are synthesized via the branched flavonoid biosynthetic pathway [11]–[13]. These secondary metabolites contribute to the 'colorful' pigmentation of flowers, fruits, seeds and leaves and are involved in several physiological and biochemical processes in plants, including UV protection, insect attraction, herbivore defense and symbiosis [14]–[16]. Plants also utilize various colors conferred by anthocyanins to recruit pollinators and to attract animals for seed dispersal [12]. Flavonoid compounds are often produced in vegetative tissues under conditions of stress, such as high light intensity, cold, nutrient deficiency, pathogen attack, or senescence [17]. As a result of this wide ranging importance, the genetics and biochemistry of the flavonoid biosynthetic pathway has been intensively studied in several plant species [18]–[20]. These studies have indicated that flavonoid composition is remarkably varied, both among plant species and even in different tissues of an individual plant [11], [21]–[23]. The flavonoid biosynthetic pathway has been extensively described in multiple publications [11]–[12], [14], [16]. The generalized scheme of the pathway is shown in Fig. 1.

To further explore the molecular basis of pigmentation in colored cotton fiber, we cloned four key genes of the flavonoid biosynthetic pathway in cotton (*GhC4H, GhCHS, GhF3*′*H*, and *GhF3*′*5*′*H*) and studied their expression profiles during the development of brown- and white cotton fibers (*Gossypium hirsutum* L.). We also analyzed the concentrations of four components of the flavonoid biosynthetic pathway, naringenin, quercetin, kaempferol and myricetin in brown- and white fibers at different developmental stages by high performance liquid chromatograph (HPLC).

## Materials and Methods

### Plant Material

Upland cotton (Xincai5, *G. hirsutum* L.) with brown fibers (Brown cotton) and its near-isogenic line (NIL) with white fibers (white cotton) were planted in an experimental field at Shihezi University (Xinjiang Autonomous Region, China) under normal agronomic management conditions. Ovules and fibers were excised carefully from developing flower buds or bolls on selected days post anthesis (every three days) and stored at –70 °C before use.

### Extraction of Total RNA

Total RNA was extracted from fibers of 18 day post anthesis (DPA) brown- or white-fiber cotton fibers using a modified CTAB method [27] and were stored at −80°C. The quality of the total RNA was verified on 1% (w/v) ethidium bromide-stained agarose gel. The double-stranded cDNAs were synthesized from total RNA using an M-MLV reverse transcriptase (Invitrogen, SuperScript™II) by an anchored oligo-dT_18_ primer according to the manufacturer's instructions.

### Cloning of flavonoid structural genes

Cotton flavonoid genes were isolated using a homologous sequence approach, based on conserved sequences of *Arabidopsis* Cinnamate-4-hydroxylase (C4H) and chalcone synthase (CHS), a *Malus* x *domestica* flavanone-3-hydroxylase (F3′H), a *Petunia* x *hybrid* flavonoid-3'5'-hydroxylase (F3′5′H) and a cDNA-AFLP differential fragment (between brown fiber and white fiber). These sequences were used as probes to match cotton ESTs in GenBank with the tBLASTn program (http://www.ncbi.nlm.nih.gov/blast). The homologous ESTs were assembled into contigs using SeqMan program of DNAStar software (DNAStar, WI, USA), and the contigs were subjected to BLASTX analysis (http://www.ncbi.nlm.nih.gov/blast) to scan for potential full-length ORFs. Subsequently, primers containing the putative ORFs were synthesized to amplify the cotton flavonoid structural genes, the cDNA derived from Xincai 5 brown fiber of 18 DPA was used as template (Table 1). The PCR products were cloned into T-cloning vector and sequenced. The predicted proteins were used to perform homology searches in GenBank using the BLASTP program (http://www.ncbi.nlm.nih.gov/blast). To further characterize these cotton flavonoid structural genes, we performed multiple alignment and phylogenetic tree analyses using the predicted proteins and their homologs via CLUSTALW [28] in DNAStar (DNAStar, WI, USA), and the phylogenetic trees were viewed by TREEVIEW [29] program.

**Table 1:** The probe sequence, ESTs and Quantitative real-time PCR primers used in this study.

Genes	Probe sequences	ESTs	Primer sequences (5'-3')
*GhC4H*	*Arabidopsis*	DT053425, DT053306,	C4H-F, GGA CCC ACC AGT TTA TTG
	C4H AM887638	DT053186, DT049233	C4H-R, ACC AGA TTA CGC TGT CCC
*GhCHS*	*Arabidopsis*	AI732033, DT051423, DT049578,	CHS-F, ATC CAG TGA AGG AGC CAT
	CHS AAZ23741	DT048936, DT048857, DT046704	CHS-R, GCT GTT TGT AAT CAT CCG
*GhF3*′*H*	*Malus x domestica*	DV437909, DT053321, DT052872,	F3'H-F, GAG AAG CTG GAC ATG GAG G
	F3'H AY965339	DT050683, DT049299	F3'H-R, TAA CCG ACC TTC GAC ACA
*GhF3*′*5*′*H*	*Petunia*	DT052722, DT051810, DT049514,	F3'5'H-F, TCA ATG CGG CCG CGG ATC
	F3'5'H ABN42195	DT048957, AI728431, AI729430	F3'5'H-R, GAA GTG TTC GTT TGG GGT TAC

### Expression analysis of the flavonoid structural genes

RNAs are extracted from roots (R), stems (S), leaves (L), petals (P) and fibers of 3, 6, 9, 12, 15, 18, 21, 24, 27 and 30 DPA of brown- and white-fiber cotton. The cDNAs were synthesized with a First-Strand cDNA Synthesis Kit (Promega) according to the manufacturer's instructions. The primers to specifically amplify PCR products of 200–300 bp from the four flavonoid structural genes were as listed Table 1. The Ubiquitin gene (UBI) was used as reference control, with the primers, 5′-CAG ATC TTC GTC AAA ACC CT-3′ and 5′-GAC TCC TTC TGG ATG TTG TA-3′ (GenBank Accession No. AI727463). Quantitative real-time PCR (QRT-PCR) were performed with the DNA Master^plus^ SYBR Green I Kit (Roche Applied Science) in a Mx3000p system (Agilent, USA). Each reaction (25 µL) contained 4 µM each primer, 2 µL cDNA(1∶100 diluted)and 10 µL PCR Buffer for Eva Green Master Mix. Thermal cycling conditions were pre-incubation 95 °C for 2 min, followed by 94 °C for 15 s, 56 °C for 20 s, and 72 °C for 20 s for 40 cycles. Each cDNA sample was run in duplicate. Relative expression ratios of target genes were calculated from the standard equation [30]. The expression assay was repeated three times and each assay was performed with three independent technical repeats. The means of the three biological experiments were calculated as the expression level of the genes.

### Measurement of naringenin, quercetin, kaempferol and myricetin by HPLC

Naringenin, quercetin, kaempferol and myricetin from brown- and white-fibers (respectively 20 g) of 3, 6, 9, 12, 15, 18, 21, 24, 27 and 30 DPA were extracted into methanol/HCl (v∶v = 99∶1) for 12 h, then extracted using supersonic waves (40 KHZ, 250 W, 30 °C) for 30 mins. The extract was centrifuged (12, 000 g for 15 mins), concentrated (to 5 ml), and filtered (0.45 µm) [31]–[32].

The four components of flavonoid biosynthesis and metabolites were determined by reverse-phase HPLC using an HP1200 system (Agilent) with a Wakosil analytical column (250 mm×34.6 mm; 5 mm packing; SGE International). HPLC separation utilized a linear solvent gradient where Solvent A was acetonitrile and Solvent B was 1% acetic (v/v with water). The gradient conditions were: 0∼16 min, 30%∼49.8% A; 16∼18 min, 49.8%∼30% A; 18∼20 min, 30% A. The column was maintained at 30 °C and the flow rate was 0.4 mL/min. Measurements of the levels of the four secondary metabolites naringenin, quercetin, kaempferol and myricetin were determined based on commercial standards (Extra synthese), and the recovery rate was tested by internal standard method [33]–[34]. Each experiment was repeated 6 times.

### Statistical analysis

Analysis of variance (ANOVA) and means were performed via the statistical software SPSS10.0.

## Results and Discussion

### Analysis of Genes Involved in Cotton Flavonoid Biosynthesis

Using a homologous sequence approach, the four flavonoid structural genes, designated: *GhC4H* (Genbank: EU921262), *GhCHS1* (Genbank: EU921263), *GhF3*′*H* (Genbank: GU062185) and *GhF3′5′H* (Genbank: GU062184) were cloned from upland cotton (Xincai5) fiber cDNAs. As shown in Table 2, the four genes encoded predicted proteins with high sequence similarity to corresponding proteins (including catalytically verified proteins) in GenBank.

**Table 2:** The proteins encoded by the flavonoid structural genes and the sequence similarity to their homologs.

								Homologous proteins							
Predicted protein	Amino acids		pI		Mw (kD)			Classes	Species		Accession Nos.		Identity(%)		
GhC4H1		505		9.06		58.02	Cinnamate-4-hydroxylase			*Populus trichocarpa*		ACC63873		92	
										*Humulus lupulus*		ACM69364		90	
										*Nicotiana tabacum*		ABC69412		90	
GhCHS		389		6.12		42.66	chalcone synthase			*Nelumbo nucifera*		ADD74168		94	
										*Vitis vinifera*		XP_002264019		93	
										*Petunia x hybrid*		BAM17286		90	
GhF3'H		510		9.12		56.76	flavanone-3-hydroxylase			*Prunus avium*		ADZ54783		75	
										*Vitis vinifera*		XP_002284165		77	
										*Malus x domestica*		ACR14867		75	
GhF3'5'H		510		9.14		57.37	flavonoid-3'5'-hydroxylase			*Populus trichocarpa*		XP_002314004		80	
										*Vitis vinifera*		BAE47007		80	
										*Camellia sinensis*		AAY23287		78	

**Figure 2:**
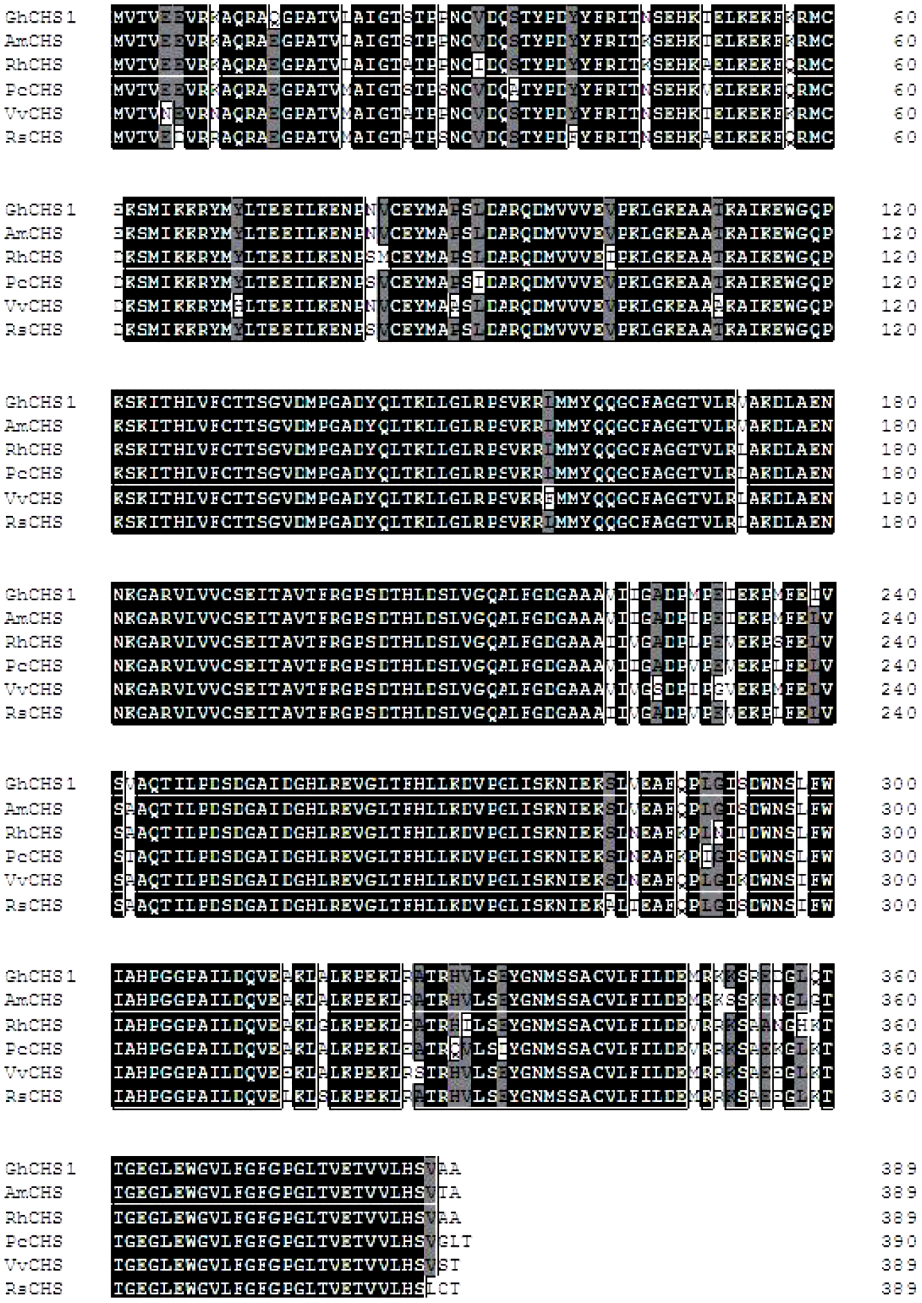
Multiple alignment of deduced amino acid sequences of plant chalcone synthase. Abbreviations of sequences RhCHS: Rosa hybrid cultivar 'Kardinal' (BAC66467); PcCHS: Pyrus communis (AAX16494); VvCHS:Vitis vinifera (BAB84111); AmCHS: Abelmoschus manihot(ACE60221); RsCHS: Rhododendron simsii (CAC88858).

**Figure 3:**
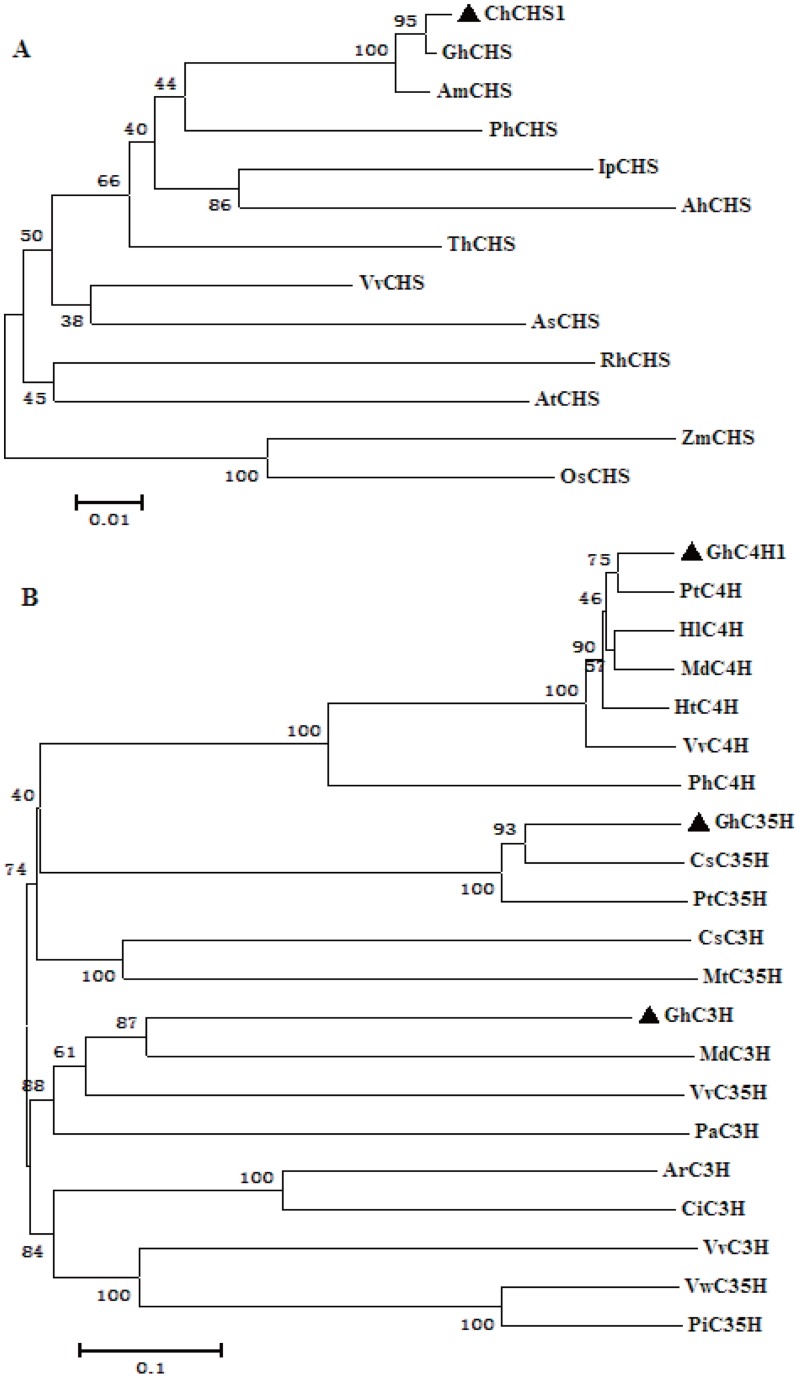
Phylogenetic analyses of chalcone synthase and cytochrome P450 superfamily proteins from various species. (A) Phylogenetic analyses of chalcone syntheses**,** (B) Phylogenetic analyses of cytochrome P450 superfamily proteins. VvCHS: *Vitis vinifera* (ABM67586), IpCHS: *Ipomoeapurpurea* (BAA20387), RhCHS: *Rud-beckia hirta* (ABN79673), ZmCHS: *Zea mays* (CAA42763), OsCHS: *Oryza sativa* (japonica cultivar-group) (BAA19186), AsCHS: *Aquilaria sinensis* (ABM73434), AtCHS; *Arabidopsis thaliana* (CAI30418), ThCHS: *Torenia hybrid cultivar* (BAB20074), AhCHS: *Arachis hypogaea* (AAO32821), AmCHS: *Abelmoschus manihot* (ACE60221); PhCHS: Petunia x hybrida (BAM17286); GhCHS: *Gossypium hirsutum* (ABS52573); PtC4H: *Populus trichocarpa* (ACC63873); HlC4H: *Humulus lupulus* (ACM69364); Ntc4h: *Nicotiana tabacum* (ABC69412); PhC4H: *Petunia x hybrid* (ADX33332); VvC4H: *Vitis vinifera* (XP_002266238); MdC4H: *Malus x domestica* (AAY87450); PaC3′H: *Prunus avium* (ADZ54783); VvC3′H: *Vitis vinifera* (XP_002284151); MdC3′H: *Malus x domestica* (ACR14867); ArC3′H: *Arabidopsis lyrata subsp. Lyrata* (XP_002871298); CsC3′H: *Camellia sinensis* (ACV74415); CiC3′H: *Cichorium intybus* (ACN65825); PtC3′5′H: *Populus trichocarpa* (XP_002314004); VvC3′5′H: *Vitis vinifera* (BAE47007); CsC3′5′H: *Camellia sinensis* (AAY23287); VwC3′5′H: *Viola x wittrockiana* (BAF93855); PiC3′5′H: *Petunia integrifolia subsp. integrifolia* (BAF34563); MtC3′5′H: *Medicago truncatula* (XP_003638760).

CHS (chalcone synthase) is a key enzyme in the biosynthesis of anthocyanin [35]. *GhCHS1* cloned from the brown cotton fibers was found to be a 1524 bp sequence containing an 1167 bp open read frame (ORF), encoding a predicted 389 amino acid polypeptide (Table 2). A multiple sequence alignment of GhCHS1 with homologous proteins revealed that it contained the conserved amino acids typical of chalcone synthase, including those at critical active sites (Cys164,His30,Ans336 and Phe215), five pocket substrate binding sites for 4-coumaroyl-CoA (Ser338,Thr197,Thr194,Glu192 and Ser133), and seven pocket sites (Thr132,Met137,Phe215,Ile254,Gly256,Phe265 and Pro375) for the cyclization reaction (Fig. 2). A phylogenetic tree constructed using 12 chalcone synthases from different species showed that the most closely related sequences to *GhCHS1* were *GhCHS* and *AmCHS* from cotton and hollyhock (both Malvaceae), respectively. The most distantly related were *OsCHS* and *ZmCHS*, from the monocots rice and maize, respectively (Fig. 3 A).

**Figure 4:**
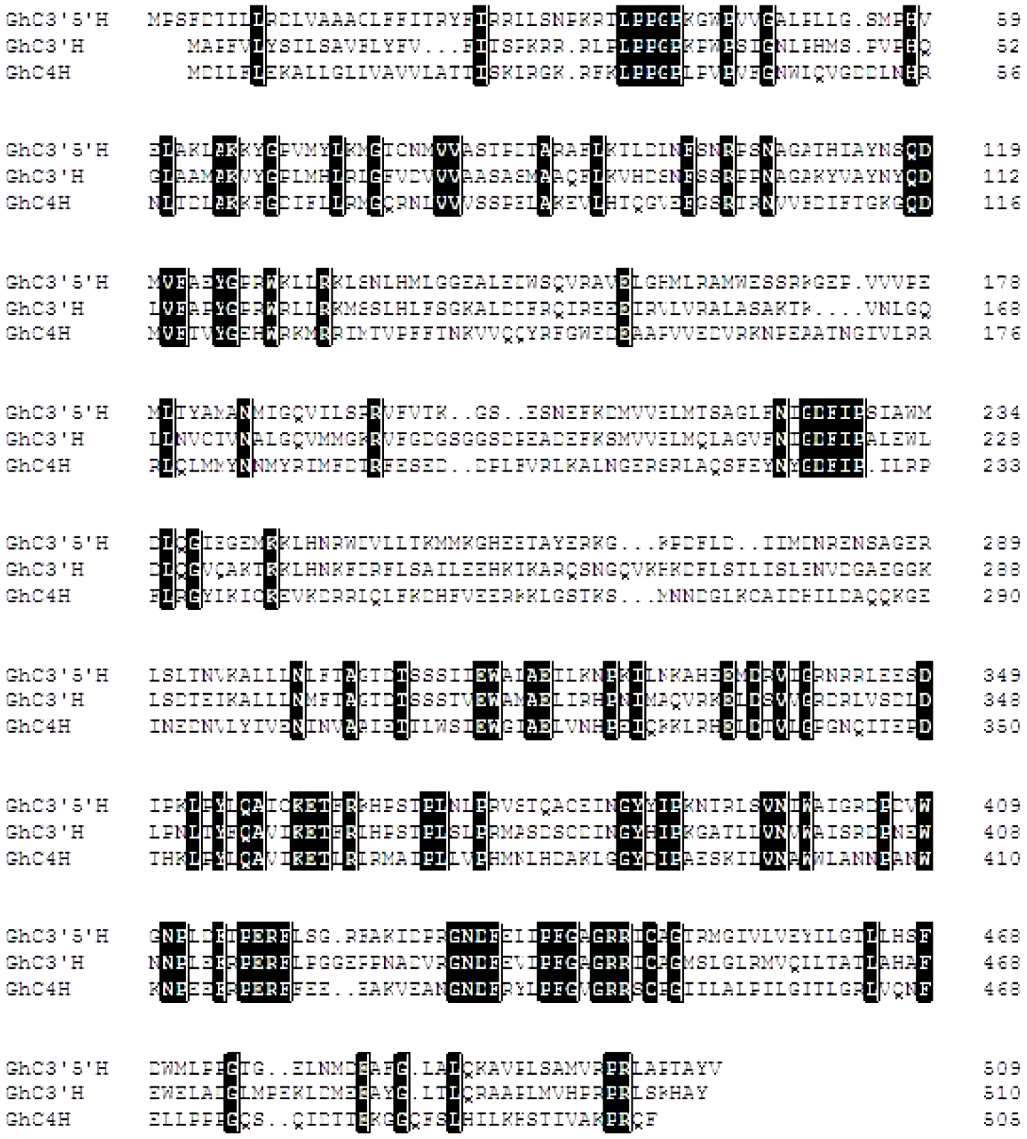
Multiple alignment of deduced amino acid sequences of Cinnamate-4-hydroxylase (C4H), flavanone-3-hydroxylase (F3′H), flavonoid-3'5'-hydroxylase (F3′5′H) from the brown cotton fiber.

The three genes, *Gh*C4H, *Gh*F3′H and *Gh*F3′5′H, belong to the cytochrome P450 superfamily, which includes other flavonoid synthetases (eg flavonol synthases, flavone synthases I, EC 1.14.11.23) and enzymes involved in other pathways such as GA_3_-oxidases, GA_2_-oxidases, and aminocyclopropane carboxylic acid oxidases [36]–[37]. Multiple sequence alignment of the predicted *GhC4H*, *GhF3*′*H* and *GhF3*′*5*′*H* proteins with their homologous counterparts (Fig. 4) revealed that the proteins contained the domains typical of members of the cytochrome P450 superfamily, including the conserved active sites, the N-terminal domain CYP motif, ‘PPGP’, and the C-terminal domain Fe-binding site, and ‘FGAGRRICAG’ that forms a structure highly conserved in different species [38]–[39]. To study the relationship of *Gh*C4H, *Gh*F3′H, *Gh*F3′5′H and other members of the family, a phylogenetic tree was constructed using 18 families of cytochrome P450s (Fig. 3 B). As shown in the phylogenetic tree, each protein family formed a unique clade, and *GhC4H*, *GhF3*′*H* and *GhF3*′*5*′*H* clustered in a clade that presumably corresponding to proteins of similar function.

### Expression profiles of cotton flavonoid structural genes

**Figure 5:**
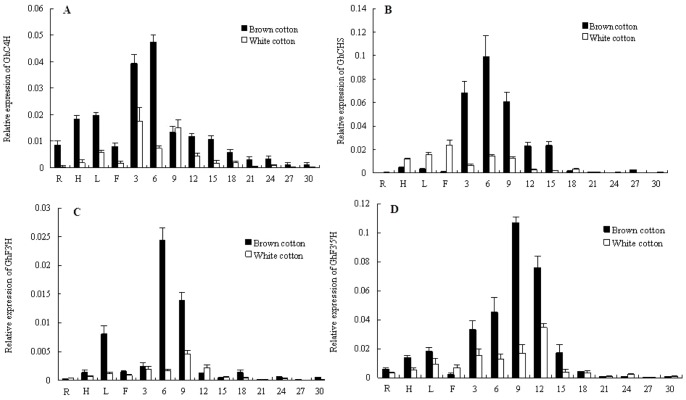
QRT-PCR analysis of four flavonoid structural genes in the brown and white cotton. A: *GhC4H1*, B: *GhCHS*, C: *GhF3*′*H*, D: *GhF3*′*5*′*H*. In the horizontal axis, 1: roots; 2: hypocotyls; 3: leaves; 4: flowers, 5–14 represent 3, 6, 9, 12, 15, 18, 21, 24, 27, 30 DPA fiber cells, respectively.

The expression of the four flavonoid structural genes, *GhC4H*, *GhCHS1, GhF3*′*H* and *GhF3*′*5*′*H* in roots (R), stems (S), leaves (L), petals (P) and fibers of 3, 6, 9, 12, 15, 18, 21, 24, 27 and 30 DPA brown and white fiber (Fig. 5) tissue was measured by QRT-PCR. All four genes were expressed, to a greater or lesser extent, in all tissues, but transcripts were considerably higher in cotton fibers. C4H is an important gene in the phenylalanine metabolic pathway, influencing many important traits through the synthesis of a large number of secondary metabolites, including lignins and flavonoids. Transcript levels for *GhC4H* were high in roots, stems, leaves and petals (Fig. 5 A). Transcript levels in the roots, stems, leaves and petals were lower for *GhCHS1*, *GhF3*′*H* and *GhF3*′*5*′*H* than for *GhC4H* (Fig. 5 B, C, D).

During cotton fiber development, transcript levels for the four genes reached a maximum between 6–12 DPA, then declined rapidly. The *GhC4H* and *GhCHS* genes had high levels of transcripts in cotton fibers from the first measurement at 3 DPA, with the levels peaking at the 6 DPA. Transcript levels for *GhF3*′*H* were high at 6 DPA, and peaked at 9 DPA. Transcript levels for *F3*′*5*′*H* peaked at 9–12 DPA. Strikingly, transcript levels for all genes were substantially higher in brown fibers than in white fibers (Fig. 5 A, B, C, D). For example, while the peak of *GhC4H* transcript levels was measured at 6 DPA in both brown and white cotton fibers, the level in brown cotton fibers was 2.5 times higher than that measured in white cotton fiber (Fig. 5 A).

Together, these data show that the flavonoid structural genes are preferentially expressed in developing fibers and considerably higher levels of expression are associated with fiber from the brown cotton (Fig. 5). The data indicate that the flavonoid pathway and these genes are involved in the development of pigmentation in brown cotton fibers [40].

### Changes of flavonoids content in developing cotton fibers

Using white cotton fiber as the control, the cumulative trend of naringenin, quercetin, kaempferol and myricetin concentrations in brown fibers were measured at different developmental stages by RP-HPLC. The recovery rates of naringenin (95.9%; *RSD%* = 1.85%), quercetin (99.2%; *RSD%* = 4.15%), kaempfero (96.3%; *RSD%* = 3.6%) and myricetin (98.7%; *RSD%* = 2.9%) were determined by comparing them with internal standards. As expected from the gene expression measurements, the concentration of the four flavonoids was significantly higher in brown fiber than in white fiber.

**Figure 6:**
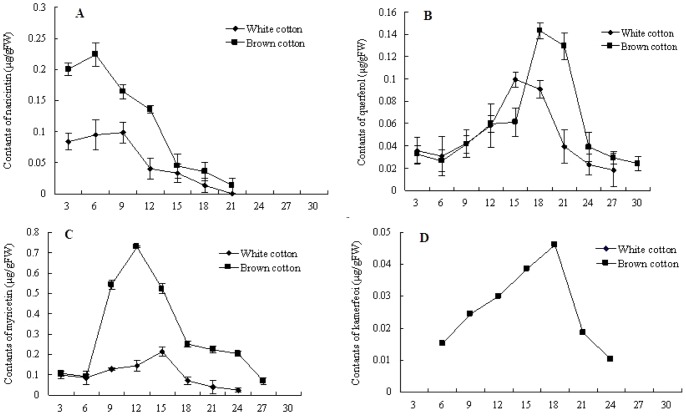
The contents of four flavonoids during the development of brown and white cotton fiber. A: naringenin, B: quercetin, C: myricetin, D: kaempferol. The numbers in horizontal axis represent 3, 6, 9, 12, 15, 18, 21, 24, 27, 30 DPA fiber cells, respectively.

These four compounds are catalyzed in the flavonoid pathway to produce stable colorless precursors that are substrates for reactions that produce the pigment substances. Naringenin is the first stable intermediate product in the flavonoid synthesis pathway, and is the substrate for most flavonoids. During development of cotton fibers, the accumulation of naringenin was considerably higher in brown fibers than in white fibers. Naringenin reached to maximum concentration at 6 DPA in both brown and white fibers, but was significantly higher in brown than in white cotton fibers (0.22 µg/gFW in brown cotton fiber, 0.09 µg/gFW in white cotton fiber) (Fig. 6 A). Commensurate with flavonoid biosynthetic pathway gene expression, naringenin was undetectable after 21 DPA, indicating that it was completely converted to other flavonoids at this time. Quercetin concentrations increased dramatically from 15 to18 DPA (Fig. 6 B). Whereas kaempferol concentrations were low in brown cotton fibers (0.05 µg/gFW (Fig. 6 D), almost none of the compound could be detected in white fibers at any stage of development. The levels of myricetin in brown fibers were high from 6- 27 DPA, increased rapidly from 6 DPA and peaked (0.8 µg/gFW) at 12 DPA. By contrast myricetin levels remained very low in white fibers throughout development. (Fig. 6 C).

As both the flavonoid structural genes and the components of the flavonoid metabolic pathway were maintained at high levels in brown fibers compared to those observed in the white fiber NIL lines, our data suggest a significant role for the flavonoid pathway in brown cotton fiber development and a strong potential that compounds produced by the pathway are involved in the pigmentation of brown fibers. Meanwhile, the naringenin, quercetin, kaempferol and myricetin were the intermediate metabolites and the precursors for leucoanthocyanidins. We would like to track and identify the end product for the fiber color on the flavonoid pathway.

### Relationship of gene expression and flavonoid accumulation in developing cotton fibers

During cotton fiber development, transcript levels for the four genes, *GhC4H*, *GhCHS1*, *GhF3*′*H* and *GhF3*′*5*′*H*, and the flavonoid levels were all higher in brown cotton fiber than they were in white cotton fibers from a NIL. The temporal pattern of transcript levels for all four genes was also consistent with timing of flavonoid accumulation in the cotton fibers. For example, *GhC4H* and *GhCHS* transcripts were high from the beginning of cotton fiber development, peaking at 6 DPA, then declining rapidly (Fig. 5 A and B). Naringenin accumulation coincided with this increase (Fig. 6 A). Furthermore, naringenin is the substrate for flavanone-3-hydroxylase (*GhF3*′*H*), and transcript levels for this gene peaked between 6–9 DPA (Fig. 5 C), potentially explaining the peak of naringenin accumulation at 6 DPA.

In this paper, we analyzed four genes and four flavonoids in the flavonoid pathway in cotton fibers. The peak of transcript levels and the trends of flavonoloid accumulation were consistent and considerably higher in brown than in white cotton fibers, indicating their particular importance in the development of brown cotton fibers.

## Conclusion

In summary, we compared the expression of genes involved in the flavonoid biosynthetic pathway in fibers from brown and white cotton (*Gossypium hirsutum L*.) using QRT-PCR, and analyzed trends in four flavonoids, naringenin, quercetin, kaempferol and myricetin at different stages of cotton fiber development by HPLC. The results showed that flavonoid structural gene expression and flavonoid accumulation were considerably higher in brown cotton fiber than in white cotton fiber, strongly suggesting that the flavonoid biosynthetic pathway contributes to the pigmentation of brown cotton fiber, This knowledge provides important information for unraveling cotton fiber pigmentation. We are confident that it is an important step in facilitating the development of new commercial cotton varieties with novel naturally colored fibres.
